# Creation of an incus recess for a middle-ear microphone using a drill or laser ablation: a comparison of equivalent noise level and middle ear transfer function

**DOI:** 10.1007/s00405-022-07532-2

**Published:** 2022-07-14

**Authors:** Robert P. Morse, Alistair Mitchell-Innes, Andreas N. Prokopiou, Richard M. Irving, Philip A. Begg

**Affiliations:** 1 School of Engineering, University of Warwick, Coventry, CV4 7AL UK; 2 ENT Department, University Hospital Birmingham, Birmingham, B15 2GW UK; 3 Institute of Translational Medicine, Birmingham, B15 2TH UK

**Keywords:** Laser Doppler vibrometry, Noise-induced hearing loss, Middle ear microphone, Equivalent noise level, Middle ear transfer function

## Abstract

**Purpose:**

Studies have assessed the trauma and change in hearing function from the use of otological drills on the ossicular chain, but not the effects of partial laser ablation of the incus. A study of the effectiveness of a novel middle-ear microphone for a cochlear implant, which required an incus recess for the microphone balltip, provided an opportunity to compare methods and inform a feasibility study of the microphone with patients.

**Methods:**

We used laser Doppler vibrometry with an insert earphone and probe microphone in 23 ears from 14 fresh-frozen cadavers to measure the equivalent noise level at the tympanic membrane that would have led to the same stapes velocity as the creation of the incus recess.

**Results:**

Drilling on the incus with a diamond burr created peak noise levels equivalent to 125.1–155.0 dB SPL at the tympanic membrane, whilst using the laser generated equivalent noise levels barely above the baseline level. The change in middle ear transfer function following drilling showed greater variability at high frequencies, but the change was not statistically significant in the three frequency bands tested.

**Conclusions:**

Whilst drilling resulted in substantially higher equivalent noise, we considered that the recess created by laser ablation was more likely to lead to movement of the microphone balltip, and therefore decrease performance or result in malfunction over time. For patients with greatly reduced residual hearing, the greater consistency from drilling the incus recess may outweigh the potential benefits of hearing preservation with laser ablation.

**Supplementary Information:**

The online version contains supplementary material available at 10.1007/s00405-022-07532-2.

## Introduction

The use of high-speed drills for removing segments of the temporal bone is ubiquitous amongst otologists [1, 2]. One risk, however, of such drilling is the proximity of the cutting edge to the ossicular chain and the potential to catch it [3] thereby transmitting energy to the inner ear with possible damage to the Organ of Corti [4], as evidenced post-operatively by pure-tone audiometry [e.g., 5] and measurement of otoacoustic emissions [e.g., 6]. With intentional drilling of the ossicular chain, for example during stapes surgery, the transmission of energy to the inner ear is assured [3, 7–9]. Laser ablation was therefore introduced to ear surgery by Sataloff [10]. Laser ablation is used for stapes surgery, both to cut the stapedius muscle and the posterior crus of the stapes and to create a recess in the footplate of the stapes [for review see 11]. Furthermore, because of the ability to cause minimal trauma to surrounding tissue with laser ablation, it has also been used directly on the ossicular chain [12] and can be used to remove cholesteatoma from the ossicular chain when attempting to preserve its transfer function [13]. Nonetheless, whilst direct trauma is reduced using laser ablation, the potential damage to the inner ear through pressure transients remains an open question [12, 14, 15]. Whether laser ablation is advantageous for other types of ear surgery is also an open question and will depend on the shape and size of cut required.

The topic is of particular relevance due to the recent development of novel active middle ear implants [e.g., 16, 17] because coupling these devices requires contact with the ossicular chain. Middle ear microphones are a potential alternative to external microphones for cochlear implants (CIs) and also require coupling [for review see 18]. One option for coupling is to create a recess within an ossicle to house a projection such as a microphone balltip. This is the approach we have adopted for our implantable cochlear implant microphone, which we refer to as a TubeMic. It consists of a microelectromechanical system (MEMS) with the diaphragm coupled to a rod and a 0.6-mm diameter ball tip that needs to be recessed into the incus body to increase the surface area of the contact point and ensure a stable connection. Before starting a feasibility study with CI patients, we wanted to address whether it would be better to create the recess using drilling or CO_2_ laser ablation in terms of preservation of the middle ear transfer function (the profile of stapes velocity across frequency), the level of noise produced by the procedure, and the ease of producing an appropriate recess. Laser ablation has several disadvantages compared with drilling, including greater cost, reduced familiarity among surgeons, and the need to adhere to laser safety precautions. We therefore considered that drilling would be the preferred option unless substantial benefits of laser ablation could be shown, in particular for preservation of middle-ear function.

Several cadaver studies have estimated the noise level at the tympanic membrane that is equivalent to sound transmission from ossicular movement caused by inadvertent ossicular contact by a drill [3, 7, 8]. We considered that the intentional drilling of the recess might require a heavier contact of the burr than for the imitation of inadvertent contact and might therefore lead to higher noise levels as well as more prolonged exposure. Alternatively, accidental contact of the drill with the ossicular chain when, for example, withdrawing the drill might lead to a greater ossicular chain movement and potentially dislocation or fracture. We therefore considered that use of a drill to create an incus recess required further study.

Although previous studies with cadavers [e.g., 15, 19] and mechanical simulations [20] have measured the equivalent noise level for laser ablation of the stapes footplate, to our knowledge the equivalent noise level has never been measured for ablation elsewhere on the ossicular chain, and in particular during the creation of an incus recess. We further considered that laser ablation might cause heat damage to the ossicular ligaments or ossicular joints, which could reduce the sensitivity of our TubeMic through reduction of the middle ear transfer function (METF). Conversely, we considered that the large potential ossicular chain movements generated whilst drilling the incus recess could stretch ossicular ligaments and the additional flexibility might also change the middle ear transfer function.

We therefore conducted a cadaveric study to measure the equivalent noise level during drilling or laser ablation of an incus recess, and to compare the middle-ear transfer function before and after creation of the recess. As described below, the middle-ear transfer function and equivalent noise were measured using laser doppler vibrometry (LDV). The final aim of the study was to enable a comparison between the ease of creating the recess with each method. The study was combined with an investigation of the effectiveness of our implantable CI microphone, which immediately followed creation of the recess. Results of this part of the investigation will be reported elsewhere.

## Materials and methods

### Surgical

Cadaveric whole heads were prepared according to guidelines from the American Society for Testing Materials (ASTM) [21] and tympanometry was performed using a Kamplex KA9 Middle Ear Analyser (PC Werth Ltd).

To gain access to the middle ear, a mastoidectomy and extended posterior tympanotomy was performed and the posterior canal wall was carefully thinned to enable good visualisation of the stapes footplate in general. When the footplate was not clearly visible, the posterior crus of the footplate was used for LDV measurement. An epitympanotomy was performed sufficiently to expose the body of the incus fully and the recess created in the incus body was central to minimise risk of shattering the bone. We measured the velocity of the stapes during formation of the recess using standard methods for LDV of the middle ear [e.g., 22, 23]. This included use of a foam tipped ear insert that was used to deliver sound and record the sound level in the ear canal. Full details of the equipment used are given in the Online Resource 1. LDV was also used when presenting acoustic tones to obtain the middle ear transfer function, which was normalized by the sound level in the ear canal. The laser beam was at an angle of 10°–50°, typically 40°, to the translational motion of the stapes footplate and measurements were cosine corrected for this angle [21].

Cadaver ears were randomly assigned to two experimental groups: in one, the incus recess was drilled using a Primado 2 drill (NSK Nakanishi Inc.); in the other group the recess was created by laser ablation with a CO_2_ Omniguide laser (FELS 25A, ARC GmBH, Germany) using the Omniguide Beampath OTO handpiece and Beampath OTO fibre. We report data for 23 ears from 14 fresh-frozen human cadaver heads designated C1, C2 etc. with a first suffix of L and R for left and right ears respectively and a second suffix of D and L denoting recess formation using a drill or laser respectively (see Online Resource 2 for full experiment parameters and cadaver demographics for each ear). We intended to collect data from 24 ears to balance conditions, but data collection from one ear was aborted because of technical problems. The age at death of the 14 cadavers ranged from 61 to 101 years old with a mean of 81 years old. When possible, both ears of the cadaver were studied with randomization for which ear was dissected first.

The diamond drill bit had a diameter of 0.6 mm, except for cadaver C6L, for which a 1-mm diameter drill was used. The cutting speed was 64,000 or 80,000 revolutions per min (rpm). Irrigation was used during drilling except for a 5-s period when the velocity of the stapes was measured using LDV. During this period, care was taken to ensure that formation of the recess was carried out without touching the cadaver, the anti-vibration table (Nexus Breadboard, Thorlabs), or the LDV/microscope (S88, Zeiss GmbH), aside from the drill contact point on the incus body. For recesses formed by laser ablation, the power of the laser was set at 3 or 4 W and used multiple pulsed bursts of 400 or 600 ms duration When simultaneously measuring stapes velocity, care was also taken to ensure that the LDV laser beam did not cross the beam for laser ablation. If necessary, a 20-gauge suction was used to clear “char” from the incus head taking care not to touch the incus where possible. The laser ablation was continued until the recess would house the balltip probe.

### Calculation of the equivalent noise level

We determined the equivalent noise level at the tympanic membrane by offline analysis of the stapes velocity recorded while the drill was in contact with the incus or during laser ablation. The duration of the analysed segments was 1 s for the drill recordings and about 0.5–1.0 s for the laser recordings; the segment duration for laser ablation varied because it was more difficult to determine when the ablation took place because the changes in velocity were close to the baseline level. Unintentionally, no equivalent noise measurements were made for C2R-D or C12L-L. We calculated the spectrum of each segment, using the discrete Fourier transform where the segments were weighted by a Bartlett window to reduce frequency leakage [24]. The magnitude spectrum was then scaled by the normalized middle ear transfer function for the cadaver to get the equivalent pressure at the tympanic membrane (or more precisely the location of the probe microphone) at each frequency, which we report as a sound pressure level in decibels. To enable comparison with results from Jiang et al. [8], we also calculated the equivalent noise level in 1/3 octave bands centred at 500 Hz, 1000 Hz, 2000 Hz, and 4000 Hz.

### METF outlier detection

The ASTM have proposed criteria [21], which we refer to as the Rosowski criteria [25] for determining whether METFs from a cadaver fall within a normative range. These were subsequently modified [23] but neither the original or modified Rosowski criteria are statistically valid because of the use of the 95% confidence interval, which relates to the precision in the estimate of the mean and not directly to the variance of individual samples from the population [25]. We have therefore rejected extreme METFs based on the median absolute deviation (MAD) around the median of METFs [26]. To ensure that the data are normally distributed (see Results), the MAD was based on the logarithm (to base 10) of the normalized velocity at each frequency. We took data points in an METF to be outliers if they were more than 3 MAD away from the median, which is considered data-conservative [26]. If, however, all data from a cadaver is rejected when one or more data points in a METF exceed the MAD criteria then it becomes highly likely that data are rejected because of the multiple comparisons. Moreover, it is not easy to correct for multiple comparisons because the correlation between each data points across frequency is not known. As a somewhat arbitrary simplification, we exclude data from a cadaver from further analysis only if the METF measured before creation of the incus recess had outliers at three or more frequencies over the frequency range.

Outlier detection was implemented in Excel 2016 (Microsoft Corporation) and the raw METF data and the analysis are given in Online Resource 2. All inferential statistical tests are for two-tailed hypotheses.

## Results

To determine whether the middle ear of individual cadavers met physiological norms, we measured the middle ear transfer function of the stapes at the start of each experiment. In common with previous studies, the METFs across individual cadavers were quite variable (see Online Resource 2), particularly above 4 kHz, but tended to have a peak at 1-kHz with a further peak at high frequencies that is likely to be due to resonance of the ear canal. The frequency of this peak varied between ears, possibly reflecting differences in ear canal volume or variations in the placement of the ear insert. Outlier rejection based on the MAD criteria given in the Methods led to data from five cadavers (C1L-L, C3L-L, C4R-D, C5R-L, and C5L-L) being rejected from further analysis; we note that excluded data tended to come from earlier experiments in the study. Eight of the twenty three ears had a peak compensated static admittance of the middle ear (“acoustic admittance “) slightly lower than the normative in vivo range (0.3–1.6 mmho) [27], i.e. they were slightly stiff, but no relation was observed between the METFs and the tympanometry measurements (see Online Resource 2 for individual tympanometry data). No obvious middle ear fluid was detected.

**Fig. 1:**
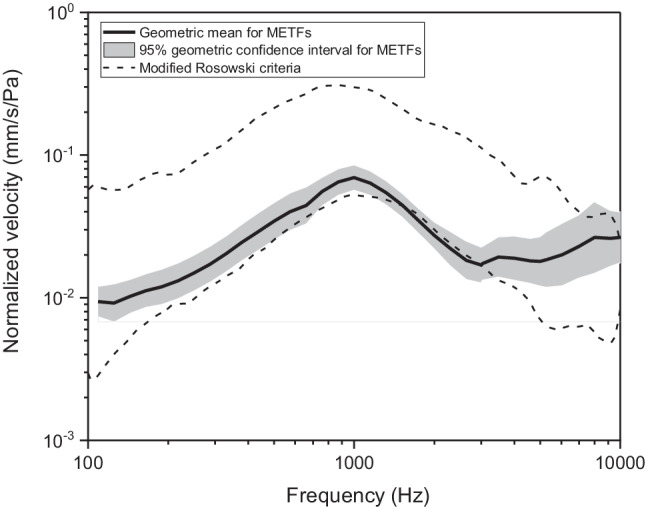
Geometric mean (thick solid line) and 95% confidence interval (shaded grey region) of the 18 METFs that satisfied the inclusion criteria. The modified Rosowski criteria [23, 25] are shown by the dashed lines

The raw METF data were not normally distributed but met the criteria for normality when a log transformation was applied (see Online Resource 2). We therefore measured the central tendency and spread at each frequency of the pooled METFs using the geometric mean and geometric 95% confidence interval rather than the arithmetic mean and arithmetic confidence interval. The geometric mean was marginally outside the modified Rosowski criteria between about 1.5 and 3 kHz but the geometric 95% confidence intervals included the criteria levels at all frequencies (Fig. 1). As discussed later, however, there was a tendency for the geometric mean of the METFs to deviate from the lower bound of the modified Rosowski criteria to the upper bound above 3 kHz; the geometric 95% confidence intervals were also larger above 3 kHz. Nonetheless, the normalized stapes velocities at the start of the study appear to be within the normative range expected and we therefore regarded the remaining cadavers to be in good physiological condition.

**Fig. 2:**
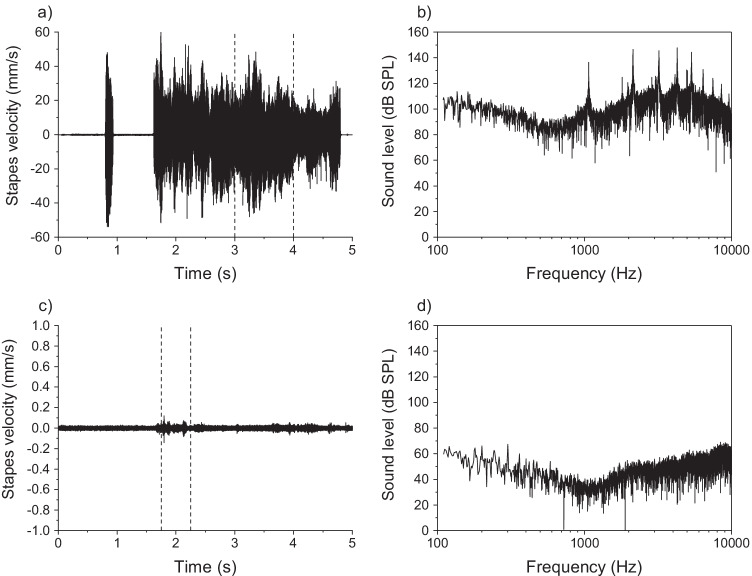
Example waveforms (left panels) and equivalent noise spectra (right panels) of Laser Doppler vibrometry recordings made during formation of an incus recess by drilling (top panels, cadaver C7R-D), or laser ablation (bottom panels, cadaver C10L-L). The waveforms show the velocity of the stapes and the equivalent noise spectra show the sound pressure level (SPL) across frequency of an acoustic signal at the position of the ear insert that would lead to equivalent energy at the stapes due to the drilling or ablation. The vertical reference lines in the plots of stapes velocity show the time windows used in the spectral analysis. Note that the waveforms for the drilling (**a**) and laser ablation (**c**) are shown on different axes

Drilling a recess into the incus at either 64,000 or 80,000 rpm, led to an increase in the stapes velocity that was substantially above the baseline level (see example for C7R-D in Fig. 2a, which shows increased velocity between about 1.5 and 5 s). During the drilling, the spectrum of the LDV signal was always periodic with a fundamental frequency of typically about 1330 Hz and had multiple harmonics (e.g., Fig. 2b). At these harmonics, the peak sound pressure level at the position of the tympanic membrane that would have led to equivalent stapes motion was substantial and ranged from 131.6 to 163.0 dB SPL with a median of 147.9 dB SPL; a Mann Whitney *U* test with 5 measurements at each speed indicated that there was no significant difference between the peak noise level for drill speeds of 64,000 and 80,000 rpm (*U* = 4.00, *p* = 0.142). In contrast, laser ablation led to at most a small increase in stapes velocity above baseline (see example for C10L-L in Fig. 2c, which shows a slight increase in velocity due to laser ablation between about 1.8 and 2.3 s), and for many cadavers it was not possible to determine from the LDV signal when ablation had occurred. The spectra of the LDV signals (e.g., Fig. 2d) were aperiodic.

**Fig. 3:**
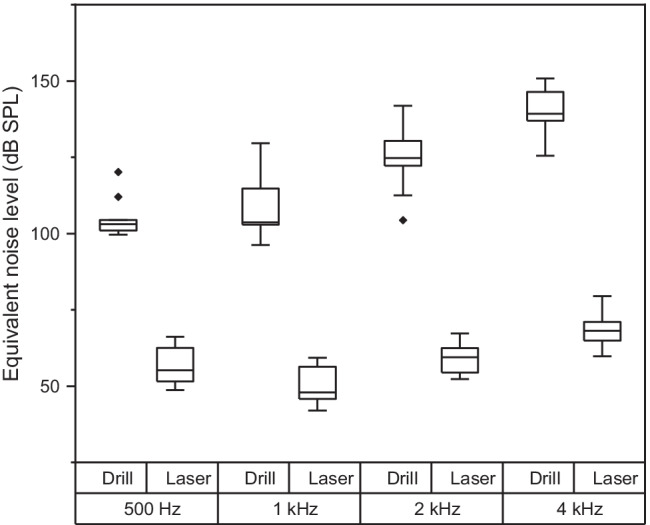
Equivalent noise level in 1/3 octave frequency bands during formation of the incus recess using a drill or laser ablation. Box whiskers are shown for frequency bands with centre frequencies at 500 Hz, 1 kHz, 2 kHz, and 4 kHz. Boxes show the 25 and 75th percentiles with the median shown by horizontal lines. The outliers outside 1.5 times the interquartile range are shown by the diamond symbol

A comparison of the equivalent noise level at the position of the ear insert is shown in Fig. 3 for four 1/3 octave frequency bands centred at 500 Hz, 1 kHz, 2 kHz, and 4 kHz. With drilling, the median sound level in each of the frequency bands was 103.1, 103.7, 124.7 and 139.2 dB SPL, respectively. These median levels were substantially greater than those measured in the corresponding bands for laser ablation, which were 58.8, 49.5, 59.4, and 68.1 dB SPL, and a Mann–Whitney *U* test indicated that the difference was significantly different in each band (*U* = 0.00, *p* < 0.001 for all bands). Given that laser ablation led to at most marginal changes to the stapes velocity, the equivalent noise measurements are largely indicative of the background noise in the dissection room. For the drilling, the equivalent noise level was greater at high frequencies and a Wilcoxon signed-rank test indicated that the level difference between the 500 Hz and 4 kHz band was significant (*Z* = − 2.666, *p* = 0.008, *r* = 0.89).

**Fig. 4:**
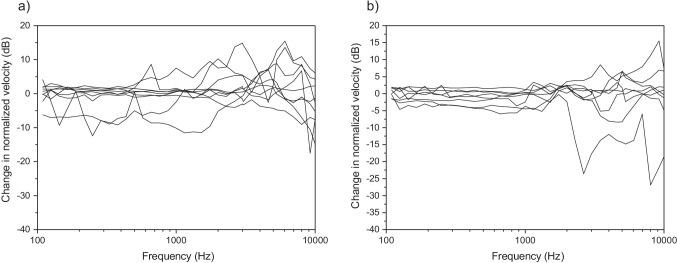
Change in the normalized velocity of the stapes for individual cadavers following formation of a recess in the incus using **a** drill and **b** laser

The change in the normalized velocity of the stapes following formation of the incus recess by a drill or laser are shown for individual cadavers in Fig. 4. Following drilling there was more than a 6 dB increase in the normalized velocity for six of the nine cadavers (C1R-D, C2R-D, C6L-D, C7R-D, C11R-D and C12R-D), and the increase was particularly notable for frequencies above 2 kHz. There was no evident pattern between the drill diameter or drill speed and the increase in stapes velocity. For frequencies below 2 kHz, there was generally no change in the normalized velocity, although for two cadavers (C1R and C6L) there was a notable increase and for two cadavers (C2R-D and C11R-D) there was a notable decrease. In contrast, the change in normalized velocity appeared to be more consistent following the formation of the recess using a laser. For all eight cadavers there was no notable change in the normalized velocity below 2 kHz. Above 2 kHz, there was a greater change in the normalized velocity for individual cadavers and three of the eight cadavers had an increase in normalized velocity above 6 dB. The proportion of cadavers, however, that had a change in normalized velocity above 100% was not significantly different for recesses made with a drill or laser (Fisher’s Exact Test, *p* = 0.347).

**Fig. 5:**
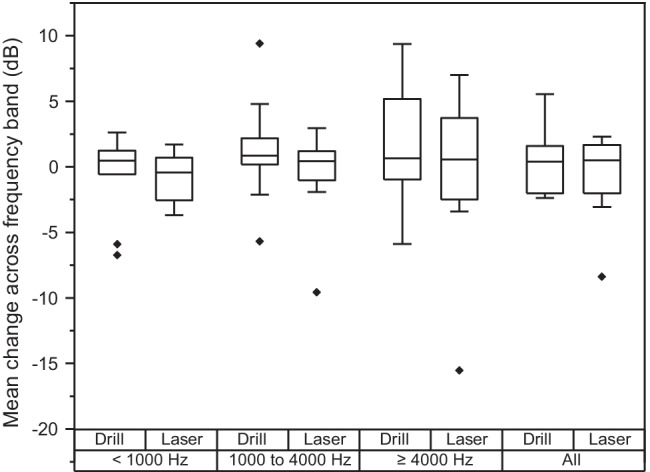
Mean change in the METFs following a recess in the incus made with a drill (*N* = 8) or laser (*N* = 10). Box whiskers are shown for all frequencies and for three sub-bands: up to 1000 Hz, 1000 Hz up to 4000 Hz, 4000 Hz and above. Boxes show the 25 and 75th percentiles with the median shown by horizontal lines. Outliers outside 1.5 times the interquartile range are shown by the diamond symbols

To enable a more in-depth statistical analysis across frequency, the change in normalized velocity for each cadaver was averaged over the whole frequency range and over three sub-bands: up to 1000 Hz, 1000 Hz up to 4000 Hz, and over 4000 Hz. As shown by the Box–Whisker plots in Fig. 5, four outliers in the sub-bands were for recesses made with the drill and three were with the laser but the median change following drilling or laser ablation were approximately the same across all frequency sub-bands. Mann–Whitney *U* tests indicated that there was no significant difference in recess creation method on the change in normalized velocity across the whole frequency range or for the three sub-bands (all frequencies: *U* = 33, *p* = 0.815; up to 1000 Hz: *U* = 29, *p* = 0.541; 1000 Hz up to 4000 Hz: *U* = 29, *p* = 0.541, and over 4000 Hz: *U* = 32, *p* = 0.743).

## Discussion

The METFs we recorded had lower normalized stapes velocity than many studies [23, 25], but below 3 kHz the METFs were comparable to those observed by Voss et al. [22] that were included in the analysis to create the original and modified Rosowski criteria [21, 23]. Above 2 kHz, our METF increased with frequency, which, whilst within the bounds of the original criteria, contrasts those published by many other groups [for comparative data see 23, 25]; we note, however, the same increase with frequency in 4 of the 6 ears in recent data from Hartl et al. [7]. Nonetheless, at 4 kHz, our mean METF is about 10 dB higher than might be expected. This increase is likely associated with the resonance of the air gap between the ear insert and the tympanic membrane. Greater variability in the response above 3 kHz is known [21, 23] and presumably explains why the ASTM criteria are limited to frequencies below 4 kHz. We were aware while conducting our study that the original and modified Rosowski criteria have been applied inappropriately [25] and this has likely led to the exclusion of possibly valid data in previous studies. Once the ear insert position was established in this study, we were therefore cautious not to adjust its position in case we inadvertently biased our recordings. We considered it appropriate to exclude ears based on their METF data in this study as described above, but we nonetheless consider it desirable that research groups pool new data so that the useful ASTM standard can be modified and used as the sole basis of exclusion in future studies. We have made our data available in Online Resource 2. The comparison of stapes mobility following formation of the incus recess used the percentage change in the transfer function and therefore was not affected by any resonance because this would have been constant during each experiment. Nonetheless, the calculation of the equivalent noise level during drilling or laser ablation used the METF of each cadaver. If our METFs are indeed about 10 dB higher than expected, then our calculation of equivalent noise level for the 4 kHz band may equivalently be about 10 dB lower than expected. This difference, however, is substantially less than the approximately 70 dB difference between the median equivalent noise levels for drilling and laser ablation for the 4 kHz band and would not therefore be expected to change the conclusion that the equivalent noise level was substantially greater for drilling.

As discussed above, our temporal bone studies showed drilling a recess in the incus can lead to a stapes velocity comparable to high intensity acoustic stimulation. Using a 0.6 mm or 1 mm diamond burr on the incus body, peak noise levels equivalent ranged from about 131.6–163.0 dB, which is similar to the range of 134 to 165 dB SPL found by Hartl et al. [7] and the range of 130 to 150 dB SPL found by Helms (1976). In common with Helms [3] and Jiang et al. [8], but not Hartl et al. we found that equivalent noise was greater in higher frequency bands.

We found no difference in the equivalent noise levels for drill speeds of 64,000 and 80,000 rpm. Data here are limited (with 5 measurements at each speed) but are consistent with Hartl et al. [7], who found no difference for drill speeds from 20,000 to 80,000 rpm on the incus, and Kylén et al. [2], who found that drill speeds from 16,000 to 25,000 rpm led to only a led to less than a 5 dB change in equivalent noise level when drilling cortical bone.

We found that when drilling with a cutting burr the recordings of stapes velocity were periodic, as evident by harmonics in the amplitude spectrum. Dalchow et al. [28] observed periodicity only with a diamond burr drill, which has irregular-sized diamond pieces attached to the steel head, but not with a cutting burr, which has a more precise shape. This periodicity with a cutting burr, however, is also evident in Fig. 5 of the study by Jiang et al. [8] with harmonics of 500 Hz although the authors do not comment on it.

The stapes velocity during laser ablation of the incus body increased marginally above the baseline measurement in some ears, but in most ears no change in velocity was observed. It is therefore very unlikely that there is any significant acoustic trauma caused by using laser ablation on the incus body with a CO_2_ laser. This agrees with previous studies for the use of CO_2_ [29] and erbium-doped yttrium aluminium garnet laser [19, 20] for stapedectomy. Nonetheless, our experience was that it was easier to create a 1-mm diameter recess that was consistently shaped and sized to house the metallic ball tip of our TubeMic by drilling than with laser ablation using the CO_2_ laser. Even the narrowest laser beam induced variable damage to surrounding bone that led to variations in the shape and size of the recess after “char” was removed. Because an inadequate recess is more likely to lead to movement of the microphone balltip, and therefore potentially cause malfunction or decrease microphone sensitivity over time because of poor coupling, we consider that the greater consistency from drilling the incus recess may outweigh the potential benefits of hearing preservation with laser ablation; this is particularly so for patients suitable for cochlear implantation.

Before the experiments we considered that the known high-noise level during drilling might cause laxity of the ossicular ligaments or joints through stretching and that laser ablation might affect the ossicular ligaments and joints through heating. Both effects could have potentially reduced the transfer function of the middle ear and would have been consequential for use of the TubeMic microphone attached to the incus as it would reduce the sensitivity of the microphone. We found, however, that although the METF increased more after drilling than laser ablation of the incus, particularly at higher frequencies, this did not reach significance. Furthermore, because the equivalent noise level for laser ablation was barely above the baseline levels this change could not be the result of trauma and the changes may have resulted from movement of the ear insert during creation of the recess; this may also have been the case for drilling. Theoretically, changes in METF post laser ablation could be caused by thermal damage to the surrounding ligaments but, given that we did not observe any increased stiffness in our data, thermal damage seems unlikely. We therefore found no evidence for choosing between drilling and laser ablation on the basis of the METF.

## Conclusions

This study, the first to directly compare the use of drilling and laser ablation on the ossicular chain, found that drilling generated higher equivalent noise levels than laser ablation but found no significant difference between using a drill or laser ablation on the METF. Whilst the noise level may be a consideration for more general middle-ear microphones or actuators, it is less so for our TubeMic, which is intended for patients who already have a severe-to-profound hearing loss. Our experience was that drilling produced a more consistent recess for the balltip of our middle-ear microphone, which was the most important consideration on our study. Moreover, given that laser ablation also has the further disadvantages of greater cost, lower accessibility, and greater inconvenience during surgery with laser precautions, we regard the reduced equivalent noise as insufficient grounds for using laser ablation to create the incus recess for our TubeMic. We therefore intend to use drilling in a field study that has been approved to test the TubeMic with CI patients.

## Supplementary Information

Below is the link to the electronic supplementary material.

Online Resource 1 is a pdf document containing further details of methods and equipment used to perform LDV

Online Resource 2 is an Excel spreadsheet containing middle-ear transfer functions that were recorded before and after creation of an incus recess using a drill or laser ablation. The spreadsheet also contains the demographics of the cadavers and the parameters for each experiment

## References

[1] Hallén O, Tjellström A (1975). The use of the drill in ear surgery. Acta Otolaryngol.

[2] Kylén P, Stjernvall J-E, Arlinger S (1977). Variables affecting the drill-generated noise levels in ear surgery. Acta Otolaryngol.

[3] Helms J (1976). Acoustic trauma from the bone cutting burr. J Laryngol Otol.

[4] Schuknecht HF, Tonndorf J (1960). Acoustic trauma of the cochlea from ear surgery. Laryngoscope.

[5] Paksoy M, Sanli A, Hardal U, Kibar S, Altin G, Erdogan BA, Bekmez ZE (2012). How drill-generated acoustic trauma effects hearing functions in an ear surgery?. Int J Head Neck Surg.

[6] Migirov L, M. W,  (2009). Influence of drilling on the distortion product otoacoustic emissions in the non-operated ear. ORL J Otorhinolaryngol Relat Spec.

[7] Hartl RMB, Mattingly JK, Greene NT, Farrell NF, Gubbels SP, Tollin DJ (2017). Drill-induced cochlear injury during otologic surgery: intracochlear pressure evidence of acoustic trauma. Otol Neurotol.

[8] Jiang D, Bibas ACS, Donnelly N, Jeronimidis G, O'Connor AF (2007). Equivalent noise level generated by drilling onto the ossicular chain as measured by laser Doppler vibrometry: a temporal bone study. Laryngoscope.

[9] Paparella MM (1962). Acoustic trauma from the bone cutting burr. Laryngoscope.

[10] Sataloff J (1967). Experimental use of laser in otosclerotic stapes. Archiv Otolaryngol.

[11] Young E, Mitchell-Innes A, Jindal M (2015). Lasers in stapes surgery: a review. J Laryngol Otol.

[12] Ilgner J, Wehner M, Lorenzen J, Bovi M, Westhofen M (2006). Morphological effects of nanosecond- and femtosecond-pulsed laser ablation on human middle ear ossicles. J Biomed Opt.

[13] Kuo CL, Liao WH, Shiao AS (2015). A review of current progress in acquired cholesteatoma management. Eur Arch Oto-Rhino-L.

[14] Stieve M, Hedrich HJ, Battmer RD, Behrens P, Muller P, Lenarz T (2009). Experimental middle ear surgery in rabbits: a new approach for reconstructing the ossicular chain. Lab Anim.

[15] Wong B, Gibbs L, Neev J, J. S,  (2000). Measurement of acoustic transients during pulsed holmium: YAG laser ablation of cadaveric human temporal bone. Lasers Surg Med.

[16] Chen T, Ren L-J, Yin D-M, Li J, Yang L, Dai PD, Zhang T-Y (2017). A comparative study of MED-EL FMT attachment to the long process of the incus in intact middle ears and its attachment to disarticulated stapes head. Hear Res.

[17] Sachse M, Hortschitz W, Stifter M, Steiner H, Sauter T (2013). Design of an implantable seismic sensor placed on the ossicular chain. Med Eng Phys.

[18] Mitchell-Innes A, Morse RRI, Begg P (2017). Implantable microphones as an alternative to external microphones for cochlear implants. Cochlear Implants Int.

[19] Keck T, Wiebe M, Rettinger G, Riechelmann H (2002). Safety of the erbium: yttrium-aluminum-garnet laser in stapes surgery in otosclerosis. Otol Neurotol.

[20] Pratisto H, Frenz M, Ith M, Romano V, Felix D, Grossenbacher R, Altermatt HJ, Weber HP (1996). Temperature and pressure effects during erbium laser stapedotomy. Lasers Surg Med.

[21] ASTM International (2014). Standard practice for describing system output of Implantable Middle Ear Hearing Devices, in Designation: F2504–05 (Reapproved 2014).

[22] Voss SE, Rosowski JJ, Merchant SN, Peake WT (2000). Acoustic responses of the human middle ear. Heart Res.

[23] Rosowski JJ, Chien W, Ravicz MA, Merchant SN (2007). Testing a method for quantifying the output of implantable middle ear hearing devices. Audiol Neurotol.

[24] Press WH, Teukolsky SA, Vetterling WT, Flannery BP (1988). Numerical recipes in C.

[25] Morse RP, Mitchell-Innes A, Prokopiou AN, Irving RM, Begg PA (2019). Inappropriate use of the “Rosowski Criteria” and “Modified Rosowski Criteria” for assessing the normal function of human temporal bones. Audiol Neurotol.

[26] Leys C, Ley C, Kleina O, Bernarda P, Licataa L (2013). Do not use standard deviation around the mean, use absolute deviation around the median. J Exp Soc Psychol.

[27] British Society of Audiology (2013) Recommended procedure for tympanometry. 26: 255–257. https://www.thebsa.org.uk/wpcontent/uploads/2013/04/OD104-35-Recommended-Procedure-Tympanometry-.pdf. 10.3109/03005369209076644 1446189

[28] Dalchow CV, Hagemeier KC, Muenscher A, Knecht R, Kameier F (2013). Investigation of noise levels generated by otologic drills. Eur Arch Oto-Rhino-L.

[29] Vernick DM (1996). A comparison of the results of KTP and CO2 laser stapedotomy. Am J Otol.

