# Supplementary material for: Creation of an incus recess for a middle-ear microphone using a drill or laser ablation: a comparison of equivalent noise level and middle ear transfer function
Source: Eur Arch Otorhinolaryngol. 2022 Jul 14;280(2):661–9. doi: 10.1007/s00405-022-07532-2 (PMC9849174; doi:10.1007/s00405-022-07532-2)
Supplement: Supplementary file 1 — Online Resource 1 is a pdf document containing further details of methods and equipment used to perform LDV [file 405_2022_7532_MOESM1_ESM.pdf]

Creation of an incus recess for a middle-ear microphone using a drill or laser ablation: A comparison of equivalent noise level and middle ear transfer function

1 Supplemental Methods

1.1 Surgery

Cadaveric whole heads were prepared within 24 hours of death and frozen at -18 ºC. Before testing, the heads were thawed for 24 hours, stored in a fridge at 5 ºC and prepared according to guidelines from the American Society for Testing Materials (ASTM) [1].

During dissection, saline was applied regularly to reduce drying of the middle ear.

1.2 Stapes velocity

We measured the velocity of the stapes during formation of the recess using LDV. LDV was also used when presenting acoustic tones to obtain the middle ear transfer function, which was normalized by the sound level in the ear canal. The LDV was performed using a OFV-534 Compact Sensor Head connected to a OPV-5000 Vibrometer Controller and VD-06 Digital High-Precision Velocity Decoder (Polytec Inc.) with a sensitivity of 50 mm/s/V giving a resolution of 0.6 µm s-1/√Hz. The sensor head was connected to a joystick operated by a micromanipulator (A-HLV-MM30, Polytec Inc.), which mounted over the lens of the operating microscope. This enabled the helium-neon laser beam (633 nm) to be directed through the opening of the mastoid and the facial recess onto an approximately 1 mm^2 of reflective tape that was placed onto the stapes footplate or posterior crus; the beam diameter was 25 µm. The laser beam was at an angle of 10 to 50°, typically 40°, to the translational motion of the stapes footplate and measurements were cosine corrected for this angle [1].

The output of the vibrometer was connected to an RSUPV-M2 audio analyser (Rohde & Schwarz Inc.), which was used to record the stapes velocity. For measurement of the stapes velocity during drilling or laser ablation, a 5-s segment of the LDV signal with a sampling frequency of 48 kHz was stored on the audio analyser and analysed offline as described below. During measurement of the middle ear transfer function (METF), the audio analyser was used to filter the LDV signal by a third-octave bandpass filter with a centre frequency locked to the stimulus frequency. The root-mean-square voltage of the filtered vibrometer signal was measured in response to each tone and normalized by the sound level of the input to get a normalized middle ear transfer [1].

Sound used during measurement of the METF was generated and controlled by the audio analyser connected to a single ER2 insert earphone (Etymotic Research Inc.) with a foam tip that was inserted into the ear canal. The stimuli were pure tones with frequencies in a geometric series from 108.8 to 10556 Hz with a base frequency of 125 Hz such that there were 5 frequencies in each decade with exact frequencies at octave intervals of the base frequency (500 Hz, 1000 Hz etc.). We also included frequencies of 3000 Hz and 5000 Hz so that we included all the frequencies in the ASTM standard [1].

The sound pressure level (SPL) in the ear canal was measured using an ER-7C Clinical Microphone System (Etymotic Research Inc.) attached to the audio analyser. The probe tube of the microphone was pushed through a hole that was made into the earphone foam tip; the probe tube extended 5 mm from the end of the tip and would have been within about 3 mm of the tympanic membrane. At the start of each experiment, the sensitivity of ER-7C, which was nominally 50 mV / Pa, was measured using the inbuilt sound generator (1-kHz tone, 94 dB SPL). During the experiment, the sound pressure level was calculated from the audio analyser input voltage and adjusted to be between 80 and 100 dB SPL over the range of stimulus frequencies.

References

1. ASTM International, *Standard practice for describing system output of Implantable Middle Ear Hearing Devices*, in *Designation: F2504 − 05 (Reapproved 2014)*. 2014: West Conshohocken, USA.
